# LncRNA LEF1-AS1 promotes metastasis of prostatic carcinoma via the Wnt/β-catenin pathway

**DOI:** 10.1186/s12935-020-01624-x

**Published:** 2020-11-10

**Authors:** Weiyuan Li, Ganggang Yang, Dengke Yang, Dong Li, Qian Sun

**Affiliations:** Department of Urology, Tongren Hospital, Shanghai Jiaotong University School of Medicine, Shanghai, 200336 People’s Republic of China

**Keywords:** LEF1-AS1, FZD2, CD44, Prostate cancer

## Abstract

**Background:**

Long noncoding RNAs (lncRNAs) are important functional regulators of many biological processes of cancers. However, the mechanisms by which lncRNAs modulate androgen-independent prostate cancer (AIPC) development remain largely unknown.

**Methods:**

Next-generation sequencing technology and RT-qPCR were used to assess LEF1-AS1 expression level in AIPC tissues and adjacent normal tissues. Functional in vitro experiments, including colony formation, EDU and transwell assays were performed to assess the role of LEF1-AS1 in AIPC. Xenograft assays were conducted to assess the effect of LEF1-AS1 on cell proliferation in vivo. Chromatin immunoprecipitation (ChIP) and RNA binding protein immunoprecipitation (RIP) assays were performed to elucidate the regulatory network of LEF1-AS1.

**Results:**

The next-generation sequencing results showed that LEF1-AS1 is significantly overexpressed in AIPC. Furthermore, our RT-qPCR assay data showed that LEF1-AS1 is overexpressed in AIPC tissues. Functional experiments showed that LEF1-AS1 promotes the proliferation, migration, invasion and angiogenic ability of AIPC cells in vitro and tumour growth in vivo by recruiting the transcription factor C-myb to the promoter of FZD2, inducing its transcription. Furthermore, LEF1-AS1 was shown to function as a competing endogenous RNA (ceRNA) that sponges miR-328 to activate CD44.

**Conclusion:**

In summary, the results of our present study revealed that LEF1-AS1 acts as a tumour promoter in the progression of AIPC. Furthermore, the results revealed that LEF1-AS1 functions as a ceRNA and regulates Wnt/β-catenin pathway activity via FZD2 and CD44. Our results provide new insights into the mechanism that links the function of LEF1-AS1 with AIPC and suggests that LEF1-AS1 may serve as a novel potential target for the improvement of AIPC therapy.

## Background

Prostate cancer (PCa) is a commonly diagnosed malignancy and the second-leading cause of cancer-related mortality in males in Western countries [1]. In its early stage, prostate cancer is often androgen dependent, although most prostate cancers can progress to exhibit androgen resistance over time [2]. AIPC carcinogenesis is a complex biological process involving a variety of genomic variations and cellular events [3]. Therefore, identifying the associated biomarkers and understanding the biology of PCa is essential for the development of effective treatment methods.

Long noncoding RNAs (lncRNAs) are a type of ncRNA (non-coding RNA) that lack protein-coding capacity and play a crucial role in regulating cell proliferation, apoptosis, migration, and invasion in addition to other cellular functions in many diseases, including cancers [4, 5]. Many lncRNAs have been shown to participate in the progression of different types of cancers, including AIPC [6]. For instance, Chakravartyet observed significantly higher lncRNA NEAT1 in tumour tissues than in adjacent tissues during AIPC progression, which significantly increased cell proliferation by regulating the epigenetic signatures of target gene promoters [7]. Human lymphoid enhancer-binding factor 1 antisense RNA 1 (LEF1-AS1), a newly discovered lncRNA located on the plus strand of chromosome 4, was previously shown to be upregulated in glioblastoma tissues, and its dysregulation was postulated to correlate with poor overall survival in patients [8, 9]. In addition, several studies have also demonstrated that LEF1-AS1 promotes cell proliferation and invasion during multiple types of cancers in recent years, suggesting that LEF1-AS1 can function as an oncogene and may be a potential therapeutic target to inhibit the progress of malignancy [10, 11]. Nevertheless, LEF1-AS1 has yet to be demonstrated to have a regulatory role associated with AIPC progression. Thus, it is necessary to elucidate the detailed function and the potential molecular mechanism of LEF1-AS1 in the regulation of AIPC progression. Increasing experimental evidence suggests that lncRNAs can sponge miRNAs and interact with proteins to regulate the expression of tumour-related genes and signalling pathways.

The Wnt/β-catenin pathway is one of the most important mechanisms responsible for regulating cell proliferation, cell polarity, migration and cell fate determination during embryonic development and in maintaining tissue homeostasis, and it has also been shown to be associated with the development of several pathologies, including cancer [12, 13]. Recently, several studies have demonstrated that the Wnt/β-catenin pathway is involved in regulating many key processes during cancer development, including in promoting cell proliferation, maintaining cancer stem cells (CSCs) and increasing metastasis [14, 15]. In the canonical Wnt/β-catenin signalling pathway, WNT ligands bind to corresponding frizzled (FZD) receptors and associated coreceptors to inhibit downstream β-catenin complex degradation, resulting in the accumulation and nuclear translocation of β-catenin, which then activates transcription through T-cell factor/lymphoid enhancer factor (TCF/LEF) [16]. Furthermore, accumulating evidence has also demonstrated that the Wnt/β-catenin signalling pathway is aberrantly activated during PCa progression [17]. Multiple molecules can activate Wnt/β-Catenin signalling during PCa progression, such as PTEN/Akt, COX-2/PGE2, PDGF, and NF-κB pathways [18–20]. However, the effects of lncRNA LEF1-AS1 on the Wnt/β-catenin pathway during AIPC progression has not been well elucidated. Therefore, understanding the underlying molecular mechanisms of the wnt/β-catenin pathway may lead to more effective diagnosis and treatment options in AIPC.

In the present study, we showed that LEF1-AS1 is upregulated in AIPC tumour tissues and promotes the proliferation, migration, invasion and angiogenic abilities of AIPC cells as well as tumour growth in vivo. Furthermore, the results of mechanistic analyses showed that LEF1-AS1 can activate the Wnt/β-catenin pathway by upregulating the expression of the Wnt/β-catenin pathway membrane receptors FZD2 and CD44. Our findings provide new insights into the molecular function of LEF1-AS1 and suggest the potential of LEF1-AS1 for use in improving therapeutic targets in AIPC treatment.

## Methods and materials

### Patients and samples

AIPC samples were obtained from 45 patients who provided informed consent in accordance with the ethical standards of the Tongren Hospital (Shanghai, China) Review Board. All samples were analysed by a pathologist and confirmed as AIPC according to histopathological evaluation. No local or systemic treatments were provided to these patients before surgery.

### Cell culture

The AIPC cell lines PC3 and DU145 were obtained from the American Type Culture Collection (Manassas, VA, USA). The normal prostate cell line RWPE was purchased from Jennio Biotech Co. (Guangzhou, China). PC3 and DU145 cells were cultured in DMEM (Gibco, Grand Island, NY, USA) supplemented with 10% heat-inactivated foetal bovine serum (FBS; Gibco). RWPE cells were cultured in RPMI 1640 medium supplemented with 10% FBS (both from Gibco). All cells were incubated at 37 °C in a humidified incubator under an atmosphere 5% CO_2_.

### Cell transfection

The LEF1-AS1 pcDNA3.3 vector was generated based on the expression vector pcDNA3.3 (Invitrogen, USA). Small interfering RNAs for LEF1-AS1, C-myb, FZD2 or CD44 (siLEF1-AS1, siC-myb, siFZD2, siCD44) and scramble siRNA (siNC) were purchased or synthetized from RiboBio (Guangzhou, China). The cells were seeded into 6-well plates and transfected with vectors using Lipofectamine 2000 (Invitrogen, Carlsbad, CA, USA) according to the manufacturer’s instructions. After incubating for 48 h, the cells were collected for further experiments.

### RNA isolation and RT-PCR analyses

Total RNA was extracted from AIPC samples and cells using TRIzol reagent (Invitrogen), and cDNA was synthesised using a QuantiTect Reverse Transcription kit (Qiagen, Valencia, CA, USA) according to the manufacturer’s protocol. Then, RT-qPCR was performed using SYBR Premix Ex Taq (Takara, Tokyo, Japan) following the manufacturer’s instructions, and the expression levels of the assayed genes were normalized to that of GAPDH. The sequences of the upstream and downstream primers used in the present study are as follows: LEF1-AS1, forward (AAGGACGAGAGAAAAGCAC) and reverse (CACACAAAGGGGAAGACC); FZD2, forward (TCGTGTACCTGTTCATCGGCAC) and reverse (CTGTGTAGAGCACGGAGAAGAC); CD44, forward (CCAGAAGGAACAGTGGTTTGGC) and reverse (ACTGTCCTCTGGGCTTGGTGTT); MMP7, forward (TCGGAGGAGATGCTCACTTCGA) and reverse (GGATCAGAGGAATGTCCCATACC); c-myc, forward (CCTGGTGCTCCATGAGGAGAC) and reverse (CAGACTCTGACCTTTTGCCAGGE); and GAPDH, forward (GTCTCCTCTGACTTCAACAGCG) and reverse ACCACCCTGTTGCTGTAGCCAA. Each experiment was repeated at least three times.

### Chromatin immunoprecipitation (ChIP)

To identify the potential transcription factors that bind to the LEF1-AS1 promoter, a ChIP assay was performed using an EZ-Magna ChIP kit (Millipore, Shanghai, China) according to the manufacturer’s protocol. In brief, cells were fixed with 4% paraformaldehyde and incubated with glycine for 10 min to generate DNA–protein cross-links. Then, the cells were lysed with cell and nuclear lysis buffers and sonicated to generate 400–800 bp chromatin fragments. The chromatin fragments were immunoprecipitated with an anti-C-myb antibody (Cell Signaling Technology, Danvers, MA, USA) or control IgG, and the resulting DNA was purified for PCR analyses All primers sequence was shown as Additional file 1: Table S1.

### RNA binding protein immunoprecipitation (RIP) assay

RIP was performed according using an EZ-Magna RIP kit (EMD Millipore, Billerica, MA, USA) following the manufacturer’s instructions. Briefly, DU145 cells grown to 80–90% confluency were lysed in complete RIP lysis buffer, and 90 μL of whole cell extract was incubated with RIP buffer containing magnetic beads conjugated with 5 μg of a human anti-C-myb antibody (Abcam) or immunoglobulin G (IgG) control. The samples were then incubated with proteinase K with shaking to digest the protein, and RNA was isolated by immunoprecipitation. Finally, the immunoprecipitated RNA was purified and analysed by RT-qPCR.

### Immunoblotting analysis

Cells (5 × 10^6^) were lysed for 20 min with lysis buffer (Beyotime Biotechnology) containing protease inhibitors (Roche, Indianapolis, IN, USA). Then, equal amounts of samples were separated by 10% sodium dodecyl sulfate polyacrylamide gel electrophoresis and transferred to polyvinylidene fluoride membranes. Subsequently, the membranes were blocked with 5% (wt/vol) skimmed milk in TBS plus Tween 20 at 4 °C overnight before being probed with antibodies against the following proteins at the indicated dilutions: GSK3β (1:1000; ab32391), p-GSK3β (1:1000; ab75814), β-catenin (1:1000; ab32572), MMP-7 (1:1000; ab271977) c-myc (1:1000; ab32072), FZD2 (1:1000; ab109094), CD44 (1:1000; ab51037) and GAPDH (1:1000; ab8245 (all obtained from Abcam, USA). The membrane-immobilized proteins were then detected using an HRP-conjugated secondary antibody (1:1000, Santa Cruz Biotech, Santa Cruz, CA).

### Colony formation assay

Colony formation assays were performed by seeding 1000 AIPC cells into each well of a 6-well plate. After culturing for 15 days, the PC3 or DU145 cells were washed with PBS and fixed in methanol for 20 min. Then, after three washes with PBS, colonies were stained with 0.1% crystal violet for 20 min and then imaged using a light microscope (Olympus, Tokyo, Japan). Clusters containing ≥ 30 cells were counted as a single colony.

### EDU assays

Cells were cultured in 96-well plates at a density of 2 × 10^3^ cells/well. Then, cell proliferation was evaluated by the EDU (5′-ethynyl-2′-deoxyuridine) incorporation assay using a KeyFluor488 Click-iT EDU kit (KeyGENBioTECH, Nanjing, China) according to the manufacturer’s instructions. Subsequently, the percentage of Edu-positive cells was calculated after fluorescence microscopy analysis.

### Cell migration and invasion assays

Cells were harvested, resuspended in serum-free medium, and then placed into the upper chamber of a transwell membrane filter (Corning, NY, USA) coated with or without Matrigel (Corning) for invasion and migration assays, respectively. After incubating for 24 h, the cells on the upper side of the filter were removed with a cotton swab. The invasive capacity of cells was evaluated by counting the invaded cells under a microscope (40 × 10). Five random fields of view were analysed for each chamber and counted using an Olympus microscope (Tokyo, Japan).

### Immunohistochemical analysis

AIPC and adjacent tissues were fixed in 4% paraformaldehyde for paraffin embedding. Then, the tissue specimens were cut into slices that were deparaffinized, rehydrated, and immersed in 3% hydrogen peroxide for 10 min to quench endogenous peroxidase activity. The cancer tissues were then immunostained for FZD2 and CD44 (Abcam, San Francisco, CA, USA; 1:100 dilution), and the signal was amplified and visualized with diaminobenzidine chromogen followed by counterstaining with haematoxylin.

### Tumourigenicity assays in nude mice

Male nude mice (6 weeks of age) obtained from the Animal Facility of Shanghai Jiao Tong University School of Medicine were used to perform tumourigenicity assays. The mice were fed sterilized food and water, and all animal experiments were approved by the Responsible Governmental Animal Ethics Committee and complied with the ARRIVE guidelines. Thirty-day-old male nude mice were subcutaneously injected in the right flank with 1 × 10^6^ AIPC cells in 200 μL of serum-free medium. Once palpable tumours were detected (after approximately 4 weeks), the mice were sacrificed, and the tumours were isolated and weighed.

### Statistical analysis

The results are presented as the means ± SD from three independent experiments performed in triplicate. The *P* values were calculated using Student *t*-test or one-way ANOVA. A *P* value of < 0.05 was considered to indicate a statistically significant result. Statistical analyses were performed using GraphPad Prism 6.0 (GraphPad Software Inc., La Jolla, California).

## Results

### LEF1-AS1 expression is upregulated in AIPC tissues and cell lines

**Fig. 1:**
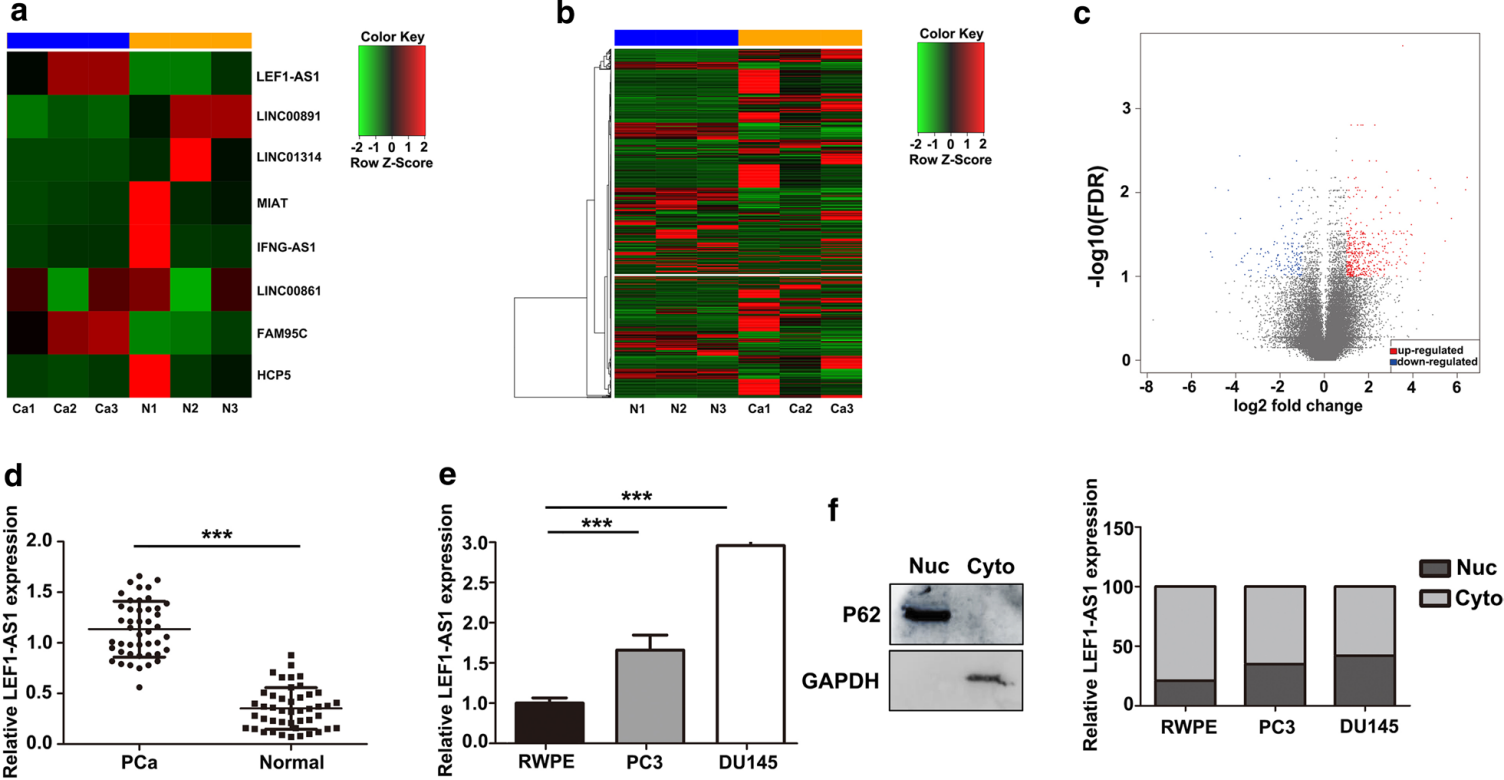
LEF1-AS1 expression is significantly higher in AIPC tissues than in adjacent tissues. **a**–**c** The heat maps and volcano plot show the expression of lncRNAs and mRNAs in AIPC tissues. **d**, **e** The RT-qPCR results showed that LEF1-AS1 expression is upregulated in AIPC tissues and cells. ***P < 0.001. **f** LEF1-AS1 localization was observed in the cytoplasm of AIPC cells, whereas minimal signal was observed in the nucleus

To elucidate the lncRNA profile and to investigate the role of lncRNAs in the development of AIPC, we performed next-generation sequencing and compared lncRNA expression in AIPC patient samples with that observed in normal samples. We identified 21 abnormally expressed lncRNAs in AIPC and observed that LEF1-AS1 was significantly increased in AIPC tissues (Fig. 1a) (Additional file 2: Table S2). In terms of mRNAs, 719 mRNAs (log2FC > 2, FDR < 0.1) were identified as being significantly differentially expressed (Fig. 1b, c). To validate the RNA sequencing results, we analysed LEF1-AS1 expression in 45 paired AIPC and adjacent tissues by RT-qPCR, the results of which confirmed the accuracy of the next-generation sequencing findings (Fig. 1d). Furthermore, LEF1-AS1 expression was increased in DU145 cells compared with PC3 and RWPE cells (Fig. 1e). These results suggested that LEF1-AS1 may function as an oncogene during AIPC progression. The results of subcellular fractionation analyses showed that the LEF1-AS1 transcript was present in the cytoplasm of AIPC cells, whereas minimal signal was observed in the nucleus (Fig. 1f), suggesting that LEF-AS1 may regulate gene expression at the transcriptional level.

### LEF1-AS1 promotes AIPC cell proliferation, migration and invasion in vitro and in vivo

**Fig. 2:**
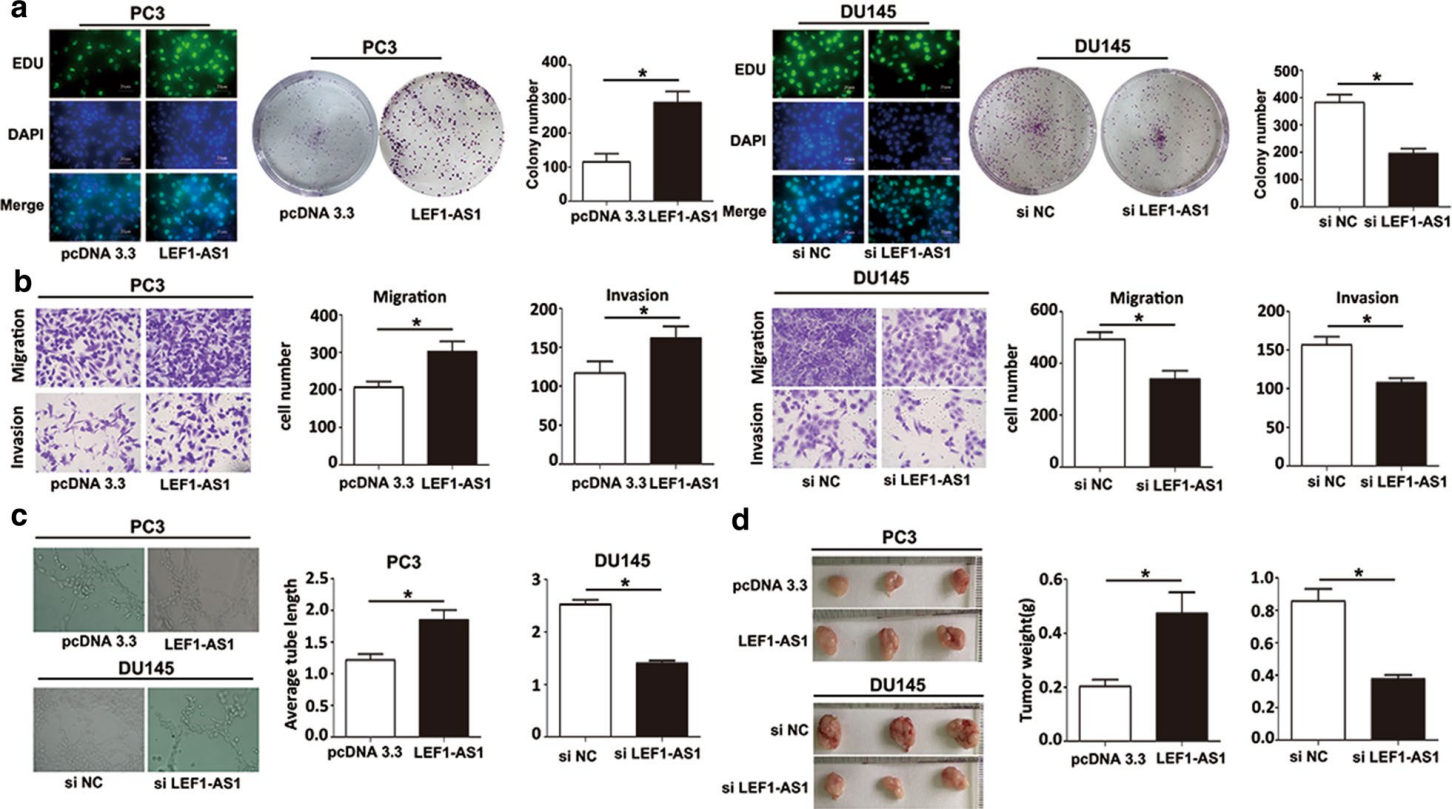
LEF1-AS1 promotes AIPC cell proliferation, migration and invasion in vitro and in vivo. **a** Colony formation and EDU assay results suggested that LEF1-AS1 overexpression can promote PC3 cell proliferation, while LEF1-AS1 knockdown could inhibit DU145 cell proliferation.*P < 0.05; scale bars: 20 μm. **b** LEF1-AS1 promoted the migratory and invasive abilities of AIPC cells. **c** The neovascularization rate was notably increased after transfection with the LEF1-AS1 overexpression plasmid and decreased after transfection with siLEF1-AS1. *P < 0.05. **d** Tumour growth was significantly decreased in the siLEF1-AS1 group in vivo. *P < 0.05

We next investigated the role of LEF1-AS1 in AIPC cell proliferation through colony formation and Edu assays. The results suggested that LEF1-AS1 overexpression could promote the proliferation of PC3 cells, while LEF1-AS1 knockdown could inhibit the proliferation of DU145 cells (Fig. 2a). Next, we evaluated AIPC cell migration and invasion using transwell-based assays. As shown in Fig. 2b, cell migration and invasion were promoted when LEF1-AS1 was overexpressed and inhibited when LEF1-AS1 was knocked down. New blood vessels are known to be essential in promoting tumour development. Therefore, we performed endothelial tube formation assays to assess the ability of LEF1-AS1 to affect the development of AIPC cells. The results demonstrated that a notably increased neovascularization rate was observed after cells were transfected with the LEF1-AS1 overexpression plasmid and decreased after transfection with siLEF1-AS1 compared to the corresponding negative controls (Fig. 2c). To further elucidate the role of LEF1-AS1 during AIPC progression in vivo, we conducted a subcutaneous tumour formation experiment in nude mice. After 4 weeks, tumour weight and volume were dramatically increased in the LEF1-AS1 overexpression group. In contrast, tumour growth, as indicated by tumour weight and volume, was significantly decreased in the siLEF1-AS1 group (Fig. 2d). Collectively, these data strongly demonstrated that LEF1-AS1 aggravated cell migration, invasion, and vasculogenic mimicry and increased cell proliferation in vivo during AIPC progression.

### LEF1-AS1 promotes Wnt/β-catenin pathway activation via frizzled class receptor 2 (FZD2)

**Fig. 3:**
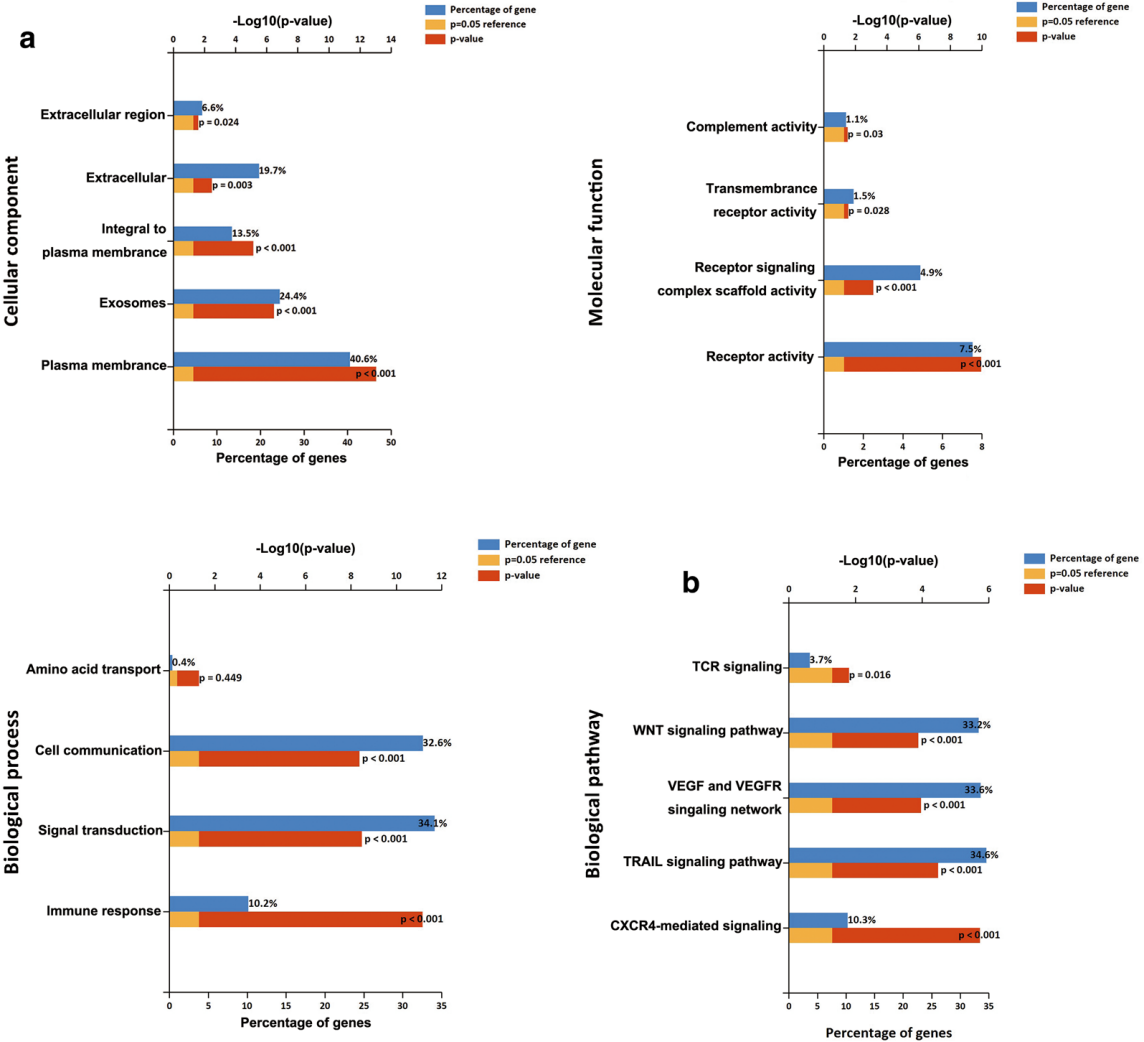
Gene ontology (GO) and KEGG pathway enrichment analyses. **a**, **b** Enriched GO terms and KEGG pathways for the upregulated genes in AIPC tissues

**Fig. 4:**
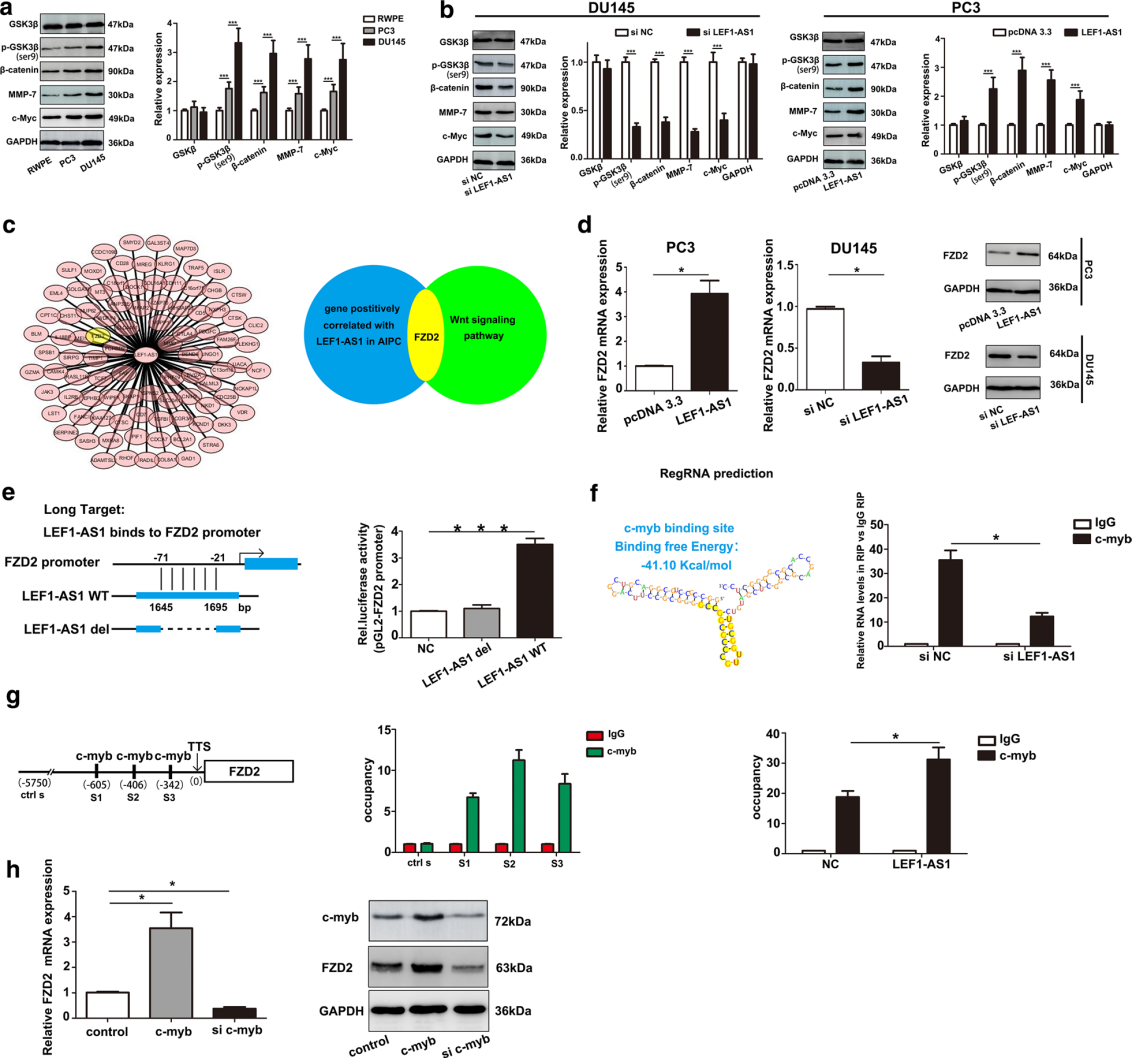
LEF1-AS1 promotes Wnt/β-catenin pathway activation via FZD2. **a** Compared to that observed in the normal cell line RWPE, Wnt/β-catenin signalling pathway activation was significantly greater in PC3 and DU145 cells. ***P < 0.001. **b** p-GSK3β (ser9), β-catenin, MMP-7 and c-myc levels were increased in PC3 cells transfected the with LEF1-AS1 overexpression plasmid and decreased in cells transfected with the siLEF1-AS1. (C) FZD2 expression was positively correlated with that of LEF1-AS1 in AIPC, as determined using the starBase 3.0 database. **d** FZD2 expression was measured in AIPC cells treated with siLEF1-AS1 or the LEF1-AS1 plasmid by RT-qPCR and western blot analysis. *P < 0.05. **e** Schematic graph of the LEF1-AS1 binding site deletion in the FZD2 promoter (predicted using LongTarget). ***P < 0.001. **f** RegRNA in silico prediction of the LEF1-AS1 secondary structure interacting with the c-myb transcription factor (protein), with a calculated binding free energy of − 41.1 kcal/mol. *P < 0.05. **g** Multiple binding sites for the transcription factor c-myb are predicted in the FZD2 promoter region (JASPAR database). The ChIP assay results confirmed the occupancy of C-myb in the FZD2 promoter. *P < 0.05. **h** FZD2 expression was measured by RT-qPCR and western blot assays. *P < 0.05

Gene ontology (GO) and KEGG pathway enrichment analysis results showed that the Wnt/β-catenin signalling pathway was one of the most enriched pathways within the upregulated mRNAs identified in AIPC, suggesting that it may be involved in the pathogenesis and development of AIPC (Fig. 3a, b). To elucidate the functions of the molecular mechanisms induced by LEF1-AS1, Wnt/β-catenin signalling pathway activity was assessed in the AIPC cell lines. Compared to that of the normal cell line RWPE, Wnt/β-catenin signalling pathway activity was significantly higher in the PC3 and DU145 cells (Fig. 4a). Subsequently, we examined the levels of GSK3β, p-GSK3β, β-catenin, MMP-7 and c-myc protein in AIPC cells after LEF1-AS1 plasmid or siLEF1-AS1 transfection. The results showed significantly higher levels of p-GSK3β (ser9), β-catenin, MMP-7 and c-myc in PC3 cells transfected with the LEF1-AS1 overexpression plasmid than that observed in the control cells. Correspondingly, in the DU145 cell lines, the levels of Wnt/β-catenin pathway proteins were decreased in cells transfected with siLEF1-AS1 (Fig. 4b). We next evaluated how LEF1-AS1 modulates the Wnt/β-catenin signalling pathway in AIPC progression. Using the starBase 3.0 database, we identified multiple genes that are positively correlated with LEF1-AS1 in AIPC, including frizzled class receptor 2 (Fig. 4c). Furthermore, as shown in Fig. 4d, significantly higher FZD2 expression was observed in cells transfected with the LEF1-AS1 plasmid than in the control cells. To further dissect the molecular mechanism by which LEF1-AS1 regulates FZD2, we analysed the sequences of LEF1-AS1 and the FZD2 promoter (+500 to −2000 bp) using the LongTarget tool. A LEF1-AS1-FZD2 binding pattern was predicted as shown in Fig. 4e, and subsequent luciferase reporter assay results demonstrated that unlike the wild-type LEF1-AS1, an LEF1-AS1 construct deleted of this binding region (LEF1-AS1 del) failed to bind the FZD2 promoter. To further investigate how LEF1-AS1 regulates FZD2 expression, we hypothesized that LEF1-AS1 may affect FZD2 transcription by affecting the ability of transcription factors to bind the FZD2 promoter. We predicted the interaction of LEF1-AS1 and C-myb via an *in-silico* analysis of regulatory RNA elements. Subsequently, RIP results further demonstrated that LEF1-AS1 RNA could be pulled down by an anti-C-myb antibody in AIPC cells, indicating the physical binding of LEF1-AS1 with C-myb (Fig. 4f). Finally, ChIP assay results confirmed the occupancy of C-myb on the FZD2 promoter. AIPC cells were subjected to ChIP with an anti-C-myb antibody, followed by RT-qPCR measurements with specific probes targeting the FZD2 promoter or −5 kb upstream (control). The results indicated that of C-myb occupancy on the FZD2 promoter region was significantly increased compared to that of the −5 kb upstream/control (Fig. 4g). The corresponding western blot assay results using ana anti-FZD2 antibody showed a similar attenuation of FZD2 protein expression upon C-myb interference (Fig. 4h). Taken together, these data suggest that LEF1-AS1 enhances FZD2 transcription by recruiting C-myb to the FZD2 promoter region.

### LEF1-AS1 increases CD44 expression by functioning as ceRNA and sponging miR-328

**Fig. 5:**
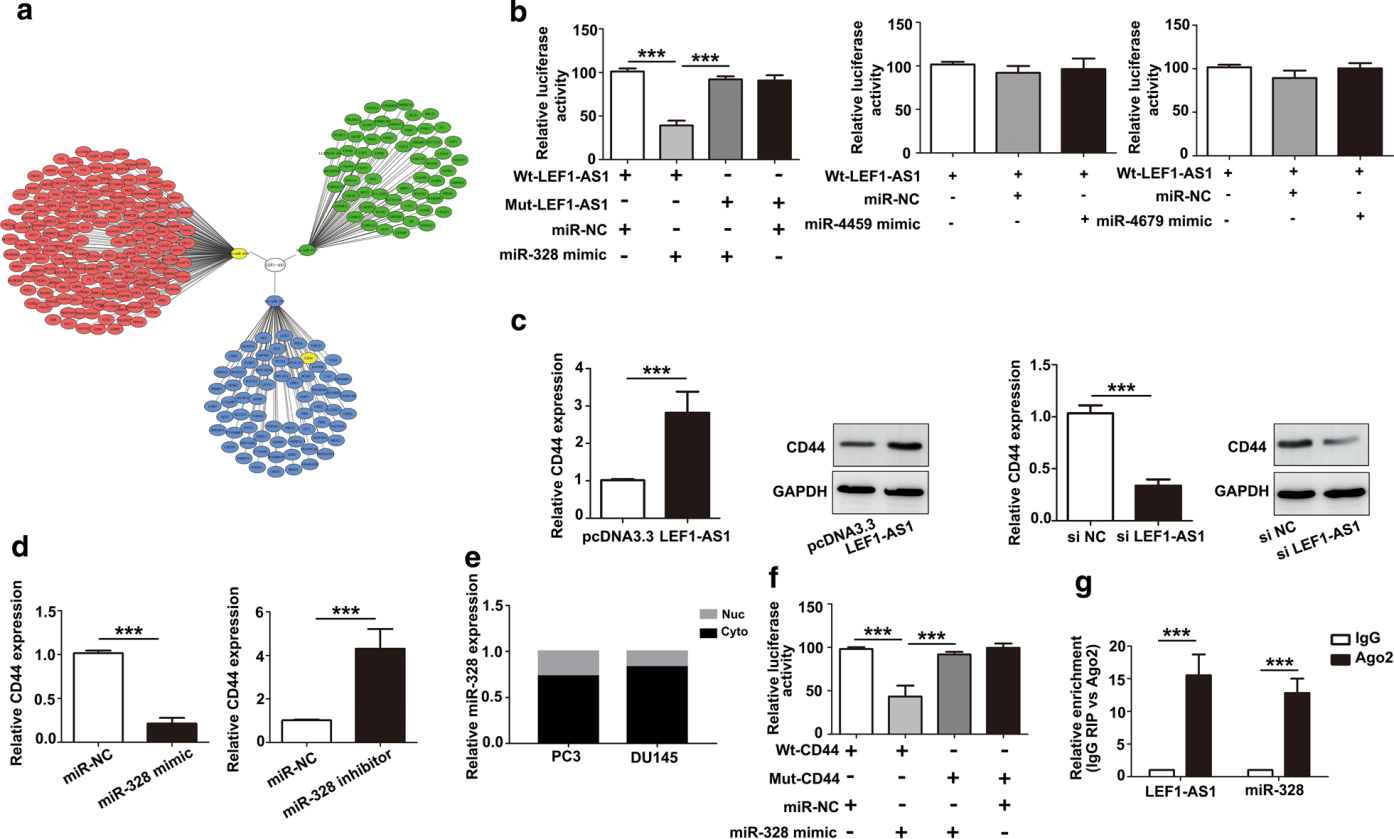
LEF1-AS1 promotes Wnt/β-catenin pathway activation via CD44. **a** Using the starBase database, we identified LEF1-AS1 and CD44 as potential targets of miR-328. **b** A dual luciferase reporter assay was performed to test whether LEF1-AS1 is a target of miR-328. ***P < 0.001. **c**, **d** CD44 expression was significantly increased by LEF1-AS1 or miR-328 inhibitor transfection. ***P < 0.001. **e** miR-328 localization was detected by Subcellular fractionation. **f** Luciferase activity was reduced in AIPC cells cotransfected with miR-328 and CD44-WT but not in cells containing CD44-Mut. ***P < 0.001. **g** The RIP assay results showed that LEF1-AS1 was enriched among Ago2-containing miRNAs compared to that observed using IgG, and miR-328 was also detected in the same precipitate. ***P < 0.001

To further study the mechanism by which LEF1-AS1 alters Wnt/β-catenin pathway activation, we used the RegRNA database and observed that LEF1-AS1 is a potential targets of 3 microRNAs (Fig. 5a). Next, we confirmed binding interactions between miR-328 with LEF1-AS1 by demonstrating a reduction in luciferase activity (Fig. 5b). Furthermore, multiple mRNAs were predicted by TargetScan and PITA, and the regulatory networks of the LEF1-AS1/miR-328 axis are shown in Fig. 5a. Among them, the CD44 gene was predicted as a downstream target gene of the LEF1-AS1/miR-328 axis. Interestingly, a previous study reported that miR-328 promotes cell proliferation by targeting CD44 in MCF-7 cells. Therefore, we assessed whether LEF1-AS1 regulates CD44 expression by sponging miR-328. As shown in Fig. 5c, d, CD44 expression was significantly increased by LEF1-AS1 or miR-328 inhibitor transfection. In contrast, CD44 expression was suppressed by the LEF1-AS1 siRNAs or miR-328 mimic. Meanwhile, the cytoplasmic localization of miR-328 was confirmed in both PC3 and DU145 cells (Fig. 5e). In addition, a dual-luciferase reporter assay was used to test whether CD44 is a direct target of miR-328. The results showed that luciferase activity was reduced in AIPC cells cotransfected with miR-328 and CD44-WT but not CD44-Mut (Fig. 5f). The ceRNA binding pattern between LEF1-AS1/CD44 and miR-328 was further validated by RNA immunoprecipitation results. As shown in Fig. 5g, significantly more RNA was enriched in the Ago2‐IP fractions of AIPC cells than in the IgG‐IP group (*P* < 0.001), indicating a direct targeting relationship between LEF1-AS1 and miR‐328. Therefore, in agreement with recent findings, the increase in LEF1-AS1 transcription appears to be an important mechanism contributing to Wnt/β-catenin pathway activation in AIPC cells by upregulating CD44.

### LEF1-AS1-mediated promotion of cell proliferation and migration is partly dependent on its regulation of FZD2 and CD44 in human prostate cancer cells

**Fig. 6:**
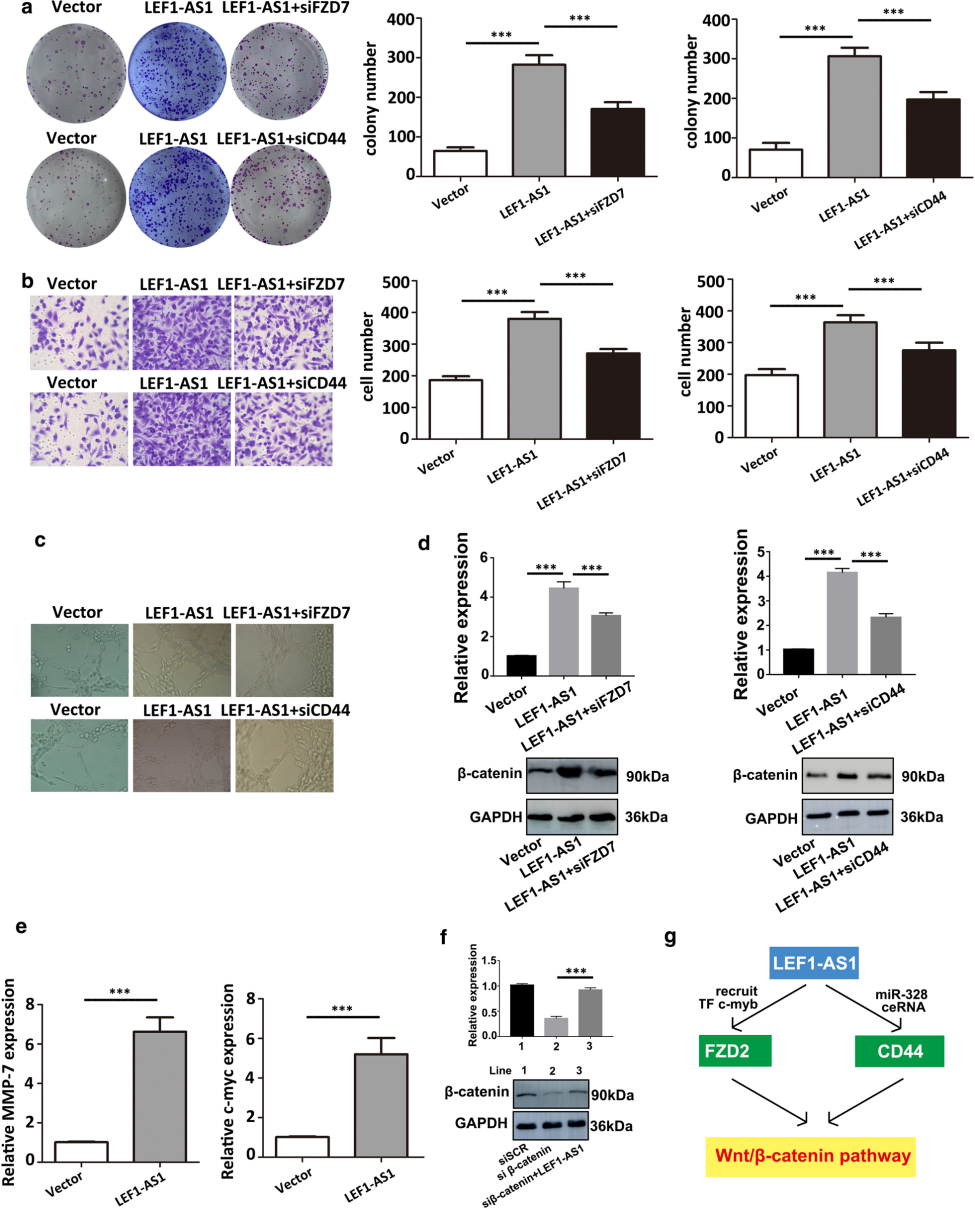
LEF1-AS1 promotes AIPC aggressiveness by targeting FZD2 and CD44. **a**–**c** Colony formation (a), transwell (b) and tube formation assays (c) were performed to assess the function of LEF1-AS1 in AIPC cells after FZD2 or CD44 knockdown. Cotransfection with LEF1-AS1 and siFZD2/siCD44 partly reversed the oncogenic effects induced by LEF1-AS1 overexpression. ***P < 0.001. **d**, **e** The expression of β-catenin and downstream targets (MMP-7 and c-myc) was also decreased after siFZD2/siCD44 transfection in LEF1-AS1-overexpressing cells compared to that observed in the control group. **f** The effect of LEF1-AS1 on the expression of β-catenin was determined by western blot. **g** Mechanism for the regulatory function of LEF1-AS1 in AIPC progression

After confirming that LEF1-AS1 regulates FZD2 and CD44 in AIPC cells, whether the oncogenic functions of LEF1-AS1 are dependent on its modulation of FZD2/CD44 expression was evaluated. Colony formation, transwell and tube formation assays were performed to assess whether FZD2 or CD44 knockdown could partly inhibit the tumour-promoting effect induced by LEF1-AS1 overexpression. The results demonstrated that the cotransfection of cells with LEF1-AS1 and siFZD2/siCD44 partly reversed the oncogenic effects induced by LEF1-AS1 overexpression (Fig. 6a–c). In addition, the expression of β-catenin and downstream targets (MMP-7 and c-myc) was also decreased after LEF1-AS1-overexpressing cells were transfected with siFZD2/siCD44 compared to that detected in the control group (Fig. 6d–e). Furthermore, LEF1-AS1 rescued the effect of siβ-catenin on AIPC cells (Fig. 6f). Taken together, as shown in Fig. 6f, our findings demonstrated that LEF1-AS1 can enhance FZD2 transcription via the recruitment of c-myb to its promoter region and increase CD44 mRNA levels by sponging miR-328, resulting in Wnt/β-catenin pathway activation.

## Discussion

Prostate cancer, a common male malignancy, is the leading cause of cancer-related mortality worldwide [21]. In recent decades, knowledge regarding the molecular mechanisms associated with PCa progression has increased, which has greatly increased the interest in identifying new molecular markers for PCa diagnosis and treatment [22].

In the present study, our next-generation sequencing and RT-qPCR results showed that LEF1-AS1 may serve as an oncogene in AIPC. The results of functional experiments showed that LEF1-AS1 promoted the proliferation, migration, invasion and angiogenic ability of AIPC cells in vitro and tumour growth in vivo. Furthermore, our chromatin immunoprecipitation (ChIP) and RNA binding protein immunoprecipitation (RIP) results showed that LEF1-AS1 functions as a competing endogenous RNA (ceRNA) that can sponge miR-328, which was shown to target CD44 and recruit the transcription factor C-myb to the FZD2 promoter to activate FZD2 transcription.

More and more efforts have been made to elucidate the molecular and cellular mechanisms of cancer progression with lncRNAs having attracted increasing attention [23]. Recently, an increasing number of studies have shown that LEF1-AS1 plays an important role in cellular proliferation, apoptosis, differentiation, and invasion during the progression of various types of cancer [24]. Zhang et al. reported that LEF1-AS1 knockdown significantly inhibits cell survival, proliferation and migration while enhancing cell apoptosis and inducing G0/G1 cell cycle arrest in oral squamous cell carcinoma, indicating a carcinogenic role of LEF1-AS1 in tumour progression [25]. Furthermore, LEF1-AS1 also has been proven to be positively correlated with lymph node metastasis and advanced stage during ovarian cancer progression, and enhanced LEF1-AS1 expression may predict a poor prognosis [26]. Nevertheless, the role of LEF1-AS1 and the underlying mechanism by which it functions in AIPC progression as remained unclear. In the present study, we performed next-generation sequencing to investigate lncRNA expression profiles and identified LEF1-AS1 as a significantly upregulated lncRNA in advanced AIPC. Subsequently, we assessed LEF1-AS1 expression levels in AIPC by RT-qPCR. The results showed that LEF1-AS1 was significantly increased during AIPC progression, which was consistent with the sequencing results. Furthermore, our results showed that LEF1-AS1 promoted the proliferation, migration, invasion and vasculogenic mimicry of AIPC cells both in vitro and in vivo. These data suggest that LEF1-AS1 acts as an oncogene during AIPC progression. Furthermore, LEF1-AS1 was shown to function as a ceRNA and regulate Wnt/β-catenin pathway through FZD2 and CD44.

Recent studies have demonstrated that Wnt/β-catenin and the downstream TCF/LEF complex, as key regulators of mitogenesis and tumourigenicity, are involved in regulating cell transformation, cell growth and cell cycle [27, 28]. Due to the frequent activation of the Wnt signalling pathway in tumour progression, FZDs play an important role in regulating this pathway, as they serve as upstream regulators of the Wnt signalling pathway. In general, FZD upregulation leads to Wnt/β-catenin pathway activation and to a lesser extent that of noncanonical pathways [29, 30]. In the present study, we confirmed that LEF1-AS1 functions in both the cytoplasm and nucleus. In the nucleus, LEF1-AS1 binds to the FZD2 promoter region. Interestingly, LEF1-AS1 also recruits the transcription factor C-myb to this region. Our results confirmed that LEF1-AS1 enhances FZD2 transcription by recruiting C-myb to its promoter region.

In the cytoplasm, LEF1-AS1 primarily serves as a sponge to regulate the expression of multiple miRNAs. For instance, a previous study reported that LEF1-AS1 functions as a ceRNA by regulating the expression of miR-544a in lung cancer [11]. In the present study, we demonstrated that LEF1-AS1 functions as a ceRNA by sponging miR-328, which leads to CD44 activation. CD44, as a multifunctional transmembrane adhesion glycoprotein, plays an important role in signal transduction. Interestingly, recent evidence has shown that CD44 expression is primarily regulated by specific signalling networks, transcription factors, and epigenetic mechanisms [31]. Furthermore, CD44 was shown to target the Wnt/β-catenin signalling pathway in tumour progression [32, 33]. In the present study, for the first time, we demonstrated that LEF1-AS1 functions as an important regulator in AIPC and regulates the activation of Wnt/β-catenin and the downstream pathway by functioning as a ceRNA to increase CD44 expression.

## Conclusion

In summary, as shown in Fig. 6g, we demonstrated that LEF1-AS1 is significantly increased during AIPC progression. Furthermore, the results of a mechanistic study showed that LEF1-AS1 functions as a ceRNA and serves as a regulator in Wnt/β-catenin pathway activation through FZD2 and CD44. Our results provide new insights into the mechanism that links the function of LEF1-AS1 with AIPC and suggests that LEF1-AS1 may serve as a novel potential target to improve AIPC therapy.

## Supplementary information

**Additional file 1: Table S1.** Primer sequences used for ChIP assays.

**Additional file 2: Table S2.** Abnormally expressed lncRNAs in AIPC and normal tissues.

## Data Availability

The analysed data sets generated during the present study are available from the corresponding author on reasonable request.
