# Supplementary material for: LncRNA LEF1-AS1 promotes metastasis of prostatic carcinoma via the Wnt/β-catenin pathway
Source: Cancer Cell Int. 2020 Nov 10;20:543. doi: 10.1186/s12935-020-01624-x (PMC7654046; doi:10.1186/s12935-020-01624-x)
Supplement: Supplementary file 1 — Additional file 1: Table S1. Primer sequences used for ChIP assays. [file 12935_2020_1624_MOESM1_ESM.docx]

| ID | Forward primer | Reverse primer |
| --- | --- | --- |
| Control S | CATGGTGGTGGACACCTGTA | TTGGGCCAGCAGAGATTCTA |
| S1 | CTCCAGAAAGAGCGGACAGA | GGGTGGGGACTCTTTAAACC |
| S2 | GAGTGAGCCAGGGTCGAGTA | ATCAGAAACCAGATGCCAACA |
| S3 | TGGGCAAACAGCCCTTAATA | GCTCTAAGCACCAACCCAAA |

Table S1. Primer sequences used for ChIP assays.
