# Supplementary material for: LncRNA LEF1-AS1 promotes metastasis of prostatic carcinoma via the Wnt/β-catenin pathway
Source: Cancer Cell Int. 2020 Nov 10;20:543. doi: 10.1186/s12935-020-01624-x (PMC7654046; doi:10.1186/s12935-020-01624-x)
Supplement: Supplementary file 2 — Additional file 2: Table S2. Abnormally expressed lncRNAs in AIPC and normal tissues. [file 12935_2020_1624_MOESM2_ESM.docx]

| gene_name | Ca_FPKM | Norm_FPKM | log2(foldchange) | pvalue | qvalue |
| --- | --- | --- | --- | --- | --- |
| LEF1-AS1 | 5.24405 | 0.23715 | 4.46681 | 5.00E-05 | 0.005485 |
| LACTB2-AS1 | 20.5134 | 4.6896 | 2.12903 | 5.00E-05 | 0.005485 |
| GS1-358P8.4 | 12.5408 | 71.5336 | -2.51199 | 5.00E-05 | 0.005485 |
| RP11-418J17.1 | 0.784688 | 0.099726 | 2.97607 | 0.0002 | 0.016596 |
| RP11-421L21.3 | 0 | 0.772715 | #NAME? | 0.0002 | 0.016596 |
| RP5-998N21.10 | 5.25587 | 1.87458 | 1.48737 | 5.00E-05 | 0.005485 |
| OR2A1-AS1 | 0.648228 | 0 | inf | 5.00E-05 | 0.005485 |
| LINC00636 | 0 | 1.11708 | #NAME? | 0.00015 | 0.013639 |
| RP11-798G7.6 | 0.864251 | 0 | inf | 0.00025 | 0.019789 |
| PTPRG-AS1 | 1.06353 | 0 | inf | 0.0004 | 0.027903 |
| RP11-192H23.7 | 3.65186 | 0.585012 | 2.64209 | 5.00E-05 | 0.005485 |
| RP11-640L9.1 | 0.119028 | 1.81073 | -3.92719 | 0.0005 | 0.032827 |
| KIAA0125 | 1.59546 | 0 | inf | 0.00025 | 0.019789 |
| PROSER2-AS1 | 0.892677 | 0 | inf | 0.00065 | 0.039916 |
| AC098820.3 | 0 | 2.33577 | #NAME? | 0.0004 | 0.027903 |
| CTB-31O20.9 | 3.84391 | 0.935367 | 2.03897 | 5.00E-05 | 0.005485 |
| CTB-193M12.3 | 43.418 | 9.51732 | 2.18967 | 0.00035 | 0.025574 |
| AC004540.5 | 2.15484 | 0.138482 | 3.95982 | 5.00E-05 | 0.005485 |
| KTN1-AS1 | 0.878515 | 0 | inf | 5.00E-05 | 0.005485 |
| CTD-2015H6.3 | 0 | 0.704591 | #NAME? | 0.0004 | 0.027903 |
| C1RL-AS1 | 170.794 | 895.447 | -2.39035 | 5.00E-05 | 0.005485 |
| RP11-214K3.20 | 0 | 1.7823 | #NAME? | 5.00E-05 | 0.005485 |
| ZNF667-AS1 | 3.52673 | 0.235901 | 3.90208 | 5.00E-05 | 0.005485 |
| RP11-326I11.4 | 118.311 | 1.40605 | 6.3948 | 5.00E-05 | 0.005485 |
| RP11-314A20.2 | 6.99184 | 0 | inf | 5.00E-05 | 0.005485 |
| LINC00667 | 0.088766 | 1.43911 | -4.01903 | 0.00035 | 0.025574 |
| RP3-333A15.2 | 4.61406 | 0.06265 | 6.20257 | 5.00E-05 | 0.005485 |
| RP11-596C23.6 | 0 | 0.793166 | #NAME? | 0.0002 | 0.016596 |
| LINC00909 | 9.19891 | 1.08677 | 3.08142 | 5.00E-05 | 0.005485 |
| RP5-837I24.6 | 2.70567 | 0.327845 | 3.0449 | 5.00E-05 | 0.005485 |
| RP11-186N15.3 | 11.1688 | 2.88936 | 1.95064 | 0.0004 | 0.027903 |
| AC018647.3 | 4.93676 | 0.743829 | 2.73052 | 5.00E-05 | 0.005485 |
| AC009133.12 | 1.05578 | 0 | inf | 5.00E-05 | 0.005485 |
| RP11-303E16.3 | 0.743308 | 0 | inf | 0.0002 | 0.016596 |
| MEF2C-AS1 | 0.138441 | 1.02743 | -2.8917 | 0.0005 | 0.032827 |
| RP11-656D10.6 | 2.42342 | 8.49997 | -1.81042 | 0.0001 | 0.009899 |
| - | 1.7362 | 0 | inf | 0.00055 | 0.035483 |

Table S2: Abnormally expressed lncRNAs in AIPC and normal tissues
